# GLUT4 translocation and dispersal operate in multiple cell types and are negatively correlated with cell size in adipocytes

**DOI:** 10.1038/s41598-022-24736-y

**Published:** 2022-11-29

**Authors:** Anna M. Koester, Angéline Geiser, Peter R. T. Bowman, Sebastian van de Linde, Nikolaj Gadegaard, Nia J. Bryant, Gwyn W. Gould

**Affiliations:** 1 Strathclyde Institute of Pharmacy and Biomedical Sciences, University of Strathclyde, Glasgow, UK; 2 Institute for Molecular, Cellular and Systems Biology, College of Medical Veterinary and Life Sciences, University of Glasgow, Glasgow, UK; 3 Department of Physics, SUPA, University of Strathclyde, 107 Rottenrow East, Glasgow, G4 0NG Scotland, UK; 4 James Watt School of Engineering, University of Glasgow, Glasgow, G12 8QQ UK; 5 Department of Biology, University of York, York, YO10 5DD UK

**Keywords:** Mechanisms of disease, Membrane trafficking

## Abstract

The regulated translocation of the glucose transporter, GLUT4, to the surface of adipocytes and muscle is a key action of insulin. This is underpinned by the delivery and fusion of GLUT4-containing vesicles with the plasma membrane. Recent studies have revealed that a further action of insulin is to mediate the dispersal of GLUT4 molecules away from the site of GLUT4 vesicle fusion with the plasma membrane. Although shown in adipocytes, whether insulin-stimulated dispersal occurs in other cells and/or is exhibited by other proteins remains a matter of debate. Here we show that insulin stimulates GLUT4 dispersal in the plasma membrane of adipocytes, induced pluripotent stem cell-derived cardiomyocytes and HeLa cells, suggesting that this phenomenon is specific to GLUT4 expressed in all cell types. By contrast, insulin-stimulated dispersal of TfR was not observed in HeLa cells, suggesting that the mechanism may be unique to GLUT4. Consistent with dispersal being an important physiological mechanism, we observed that insulin-stimulated GLUT4 dispersal is reduced under conditions of insulin resistance. Adipocytes of different sizes have been shown to exhibit distinct metabolic properties: larger adipocytes exhibit reduced insulin-stimulated glucose transport compared to smaller cells. Here we show that both GLUT4 delivery to the plasma membrane and GLUT4 dispersal are reduced in larger adipocytes, supporting the hypothesis that larger adipocytes are refractory to insulin challenge compared to their smaller counterparts, even within a supposedly homogeneous population of cells.

## Introduction

The insulin-dependent increase in glucose transport in peripheral tissues (muscle, adipose) is a key action of insulin^1,2^. It is now well-established that this effect is underpinned by a unique glucose transporter, GLUT4, expressed in insulin-sensitive cells. In the absence of insulin, GLUT4 is retained intracellularly through active sequestration in multiple intracellular locations, referred to as the GLUT4 storage compartment (GSC); a subset of these vesicles, termed ‘insulin responsive vesicles’ are mobilised to the plasma membrane in response to insulin where they dock and fuse, delivering GLUT4 to the plasma membrane and thus accounting for increased rates of glucose transport into these tissues^3–6^.

Our understanding of insulin-stimulated glucose transport has been enhanced by the application of imaging approaches which have provided new insight into the complexity of GLUT4 containing vesicle movement. Under basal conditions GLUT4 containing vesicles moved along predefined trajectories over the plasma membrane, exhibiting periodic tethering. Insulin stimulation resulted in a drastic reduction of GLUT4 containing vesicle movement indicating immobilization of these vesicles at the plasma membrane, presumably prior to fusion^7–12^.

Many membrane proteins have been shown to form nanoscopic aggregates^13–16^. Compared to their monomeric counterparts, clustered membrane proteins are thought to exhibit novel properties. One example is that protein clusters transduce cellular signals like digital circuits with improved signal-to-noise performance than monomers, which are comparable to analogue units^16,17^. Hence, understanding the behaviour of membrane proteins is likely to underpin insight into their regulation. While much detail of the molecular mechanism of GLUT4 translocation has been revealed, recent work has indicated that the organisation of GLUT4 within the plasma membrane is also regulated by insulin and that this may be impacted by disease. Using time-lapse total internal reflection fluorescence (TIRF) microscopy of GLUT4-GFP in primary adipocytes, GLUT4 near the plasma membrane was found either localised within relatively stationary clusters or in mobile structures thought to be GSCs adjacent to the plasma membrane^18^. High resolution imaging of surface-exposed GLUT4 inserted into the plasma membrane using antibodies directed against the exofacial HA epitope confirmed that GLUT4 distribution within the plasma membrane is inhomogeneous, being identified both in clustered domains and as a diffuse staining^18,19^. The authors identified two distinct modes of GLUT4-vesicle exocytosis. In the first, termed ‘fusion-with-release’, GLUT4 is dispersed within the plasma membrane. By contrast, so-called ‘fusion-with-retention’ events retain GLUT4 molecules at the fusion site (‘clustered’). In the absence of insulin, most fusion events were fusion-with-retention. Strikingly, insulin was shown to result in a ~ 60-fold increase in fusion-with-release events within 2–3 min^18^. Importantly these effects were also identified using the exofacial HA-epitope within HA-GLUT4-GFP expressed in 3T3-L1 adipocytes, indicating that insulin results in dispersal of GLUT4 within the plasma membrane^20^. It is now believed that insulin has three effects on plasma membrane localised GLUT4 molecules: insulin increases the fraction of dispersed GLUT4 upon its delivery to the plasma membrane, increases the dissociation of GLUT4 monomers from clusters and decreases the rate of GLUT4 endocytosis^7^ (although the latter point may not be a universal feature of insulin action–see^6,22,23^). This has been further supported using imaging methods which focus exclusively on GLUT4 inserted into the plasma membrane^20^,^21^.

Evidence for a functional role of GLUT4 dispersal in the control of glucose transport has been provided by our recent demonstration that impairment of insulin-stimulated GLUT4 dispersal is accompanied by reduced insulin-stimulated glucose transport^21^. Furthermore, insulin resistance was found to induce a more clustered distribution of GLUT4, with more molecules per cluster^20^, implying that the regulation of post-fusion GLUT4 distribution may be impaired in disease^24^. Multiple defects in GLUT4 trafficking have been identified in insulin resistance/Type 2 diabetes, including reduced translocation^25^ and impaired GLUT4 vesicle tethering^24^. The observation that GLUT4 dispersal is also a potential point of dysregulation emphasises the multifactorial nature of insulin resistance and adds weight to the need to understand this process.

In this study we utilised the exofacial HA-epitope within HA-GLUT4-GFP in combination with direct Stochastic Optical Reconstruction Microscopy (dSTORM) analysis in a range of cell types to test the hypothesis that GLUT4 dispersal was a phenomenon found in all insulin-sensitive cells. We show that insulin-stimulated GLUT4 dispersal can be observed in 3T3-L1 adipocytes, HeLa cells and induced pluripotent stem cell-derived cardiomyocytes. By contrast the transferrin receptor, a prototypical recycling endosomal protein, does not exhibit insulin-stimulated dispersal. We confirm previous studies showing reduced dispersal in a model of insulin resistance, and further show that both insulin-stimulated GLUT4 delivery to the plasma membrane and insulin-stimulated GLUT4 dispersal are negatively correlated with cell size in adipocytes.

## Materials and methods

### Cell lines, plasmids and viral infections

Stable lines of 3T3-L1 fibroblasts and HeLa cells expressing HA-GLUT4-GFP had previously been generated in the lab^26^ from stocks purchased from ATCC and screened routinely to confirm the absence of mycoplasma. Cells were incubated in a 10% CO_2_ humidified atmosphere at 37 °C and grown and differentiated as described^27^.

Induced pluripotent stem cell-derived cardiomyocytes (iPSC-CMs) were commercially obtained directly from NCardia (#Ax-B-HC02-1 M) and Cellular Dynamics International (#CMC-100–012-000.5) and used as outlined^28^. iPSC-CMs were plated at a density of 35,000 or 50,000 viable cells per well according to manufacturer’s instructions into wells of a 96-well plate coated with fibronectin from bovine plasma (Sigma) at a final concentration of ~ 2 µg/cm^2^. Cells were maintained in a sterile humidified incubator (37 °C, 5% CO_2_) in the appropriate maintenance medium provided by the manufacturers, which was replaced 4–24 h post plating, and every 24–48 h thereafter. CDI iPSC-CMs were infected with HA-GLUT4-GFP adenovirus (commercially prepared by ViraQuest Inc., Iowa) from plasmids described^26,29,30^ at an MOI of 50:1. Infection was assessed before fixation by checking the GFP signal using epifluorescence. In some experiments, cells were infected with a lentivirus designed to express full-length wild-type human GLUT4.

2-deoxy-D-glucose uptake in iPSC-CMs or 3T3-L1 adipocytes was performed exactly as described^27,28^. The GFP-tagged transferrin receptor (TfR-GFP) was from Jennifer Lippincott-Schwartz (Janelia, VA).

### Hyperinsulinemia model of insulin resistance

3T3-L1 adipocytes at day 10 post differentiation were incubated with 500 nM insulin for 24 h as outlined^31,32^. After this time, cells were washed four times with Krebs–Ringer MES (136 mM NaCl, 4.7 mM KCl, 1.25 mM CaCl_2_, 1.25 mM MgSO_4_, 10 mM MES, pH 6), three times with Krebs–Ringer phosphate (136 mM NaCl, 4.7 mM KCl, 1.25 mM CaCl_2_, 1.25 mM MgSO_4_, 10 mM NaH_2_PO_4_/Na_2_HPO_4_, pH 7.4) and glucose transport assayed as outlined with or without a subsequent acute insulin challenge (100 nM insulin, 20 min).

### Analysis of GLUT4 dispersal using dSTORM

dSTORM measurements were performed in fixed cells using anti-HA to identify GLUT4 localised within the plasma membrane. Prior to measurement, all cells were starved of serum for 2 h. 3T3-L1 adipocytes and iPSC-CMs were stimulated with 100 nM insulin whereas HeLa cells were stimulated with 1 μM insulin for 20 min or left untreated. Subsequently, cells were fixed with 4% paraformaldehyde in PBS overnight at 4 °C. The samples were quenched with 50 mM NH_4_Cl in PBS for 10 min at room temperature, washed with PBS then incubated in blocking solution (2% BSA with 5% goat serum in PBS) for 30 min. Afterwards cells were incubated with a conjugated anti-HA antibody coupled to Alexa Fluor 647 at a concentration of 8 mg/mL in blocking solution for 1 h in the dark. Samples were washed with PBS for 10 min 3 times on an orbital shaker.

### dSTORM image acquisition and reconstruction

The dSTORM image sequences were acquired as described in^33^. In brief, images were collected in TIRF configuration using 647 nm laser light at 100% power (150 mW), on an Andor iXon 897 EMCCD camera using a centred 256 × 256-pixel region at 30 ms exposure for 10,000 frames and a gain of 200. The dSTORM data were processed using the ImageJ plugin ThunderSTORM^34^. The image reconstruction parameters chosen are lined out in the following: pre-detection wavelet filter (B-spline, scale 2, order 3), initial detection by local maximum with 8-connected neighbourhoods (radius 1, threshold at two standard deviations of the F1 wavelet), and sub-pixel localisation by integrated Gaussian point-spread function (PSF) and maximum likelihood estimator with a fitting radius of 3 pixels. The first pass detected localisations were filtered according to the following criteria: an intensity range of 500–5000 photons, a sigma range of 25–250, and a localisation uncertainty of less than 25 nm. Subsequently, the filtered data set was corrected for sample drift using cross-correlation of images from 5 bins at a magnification of 5. Repeated localisations, such as can occur from dye re-blinking, were reduced by merging points which re-appeared within 20 nm and 1 frame of each other.

#### HDBSCAN analysis

The HDBSCAN library was downloaded from Github. ROI of 4 μm × 4 μm were selected in ImageJ for each cell and GLUT4 molecule coordinates were processed using code written in house using min_cluster_size = 5 and min-samples = 30 to provide clear visualisation of the dataset^35–37^.

#### Spatial statistics analysis with SR Tesseler

We analysed the spatial point patterns of GLUT4 and TfR molecule coordinates with the freely available, open-source SR-Tesseler software^38^ downloaded from Github (https://github.com/flevet/SR-Tesseler). Firstly, molecules were segmented using Voronoï tessellation. SR Tesseler calculates Voronoï diagrams by subdividing space into polygonal regions centred around a given set of points (molecule localizations in this case) in terms of Euclidian distance. Polygons are directly influenced by their neighbours, and first rank polygon density is statistically calculated. The polygon density distribution is plotted on a logarithmic scale and divided by the average density of the whole data set. By comparing the localization distribution to a reference distribution that is spatially uniform (similarly to Monte Carlo simulations) a density threshold is automatically defined and polygons within the defined thresholds are selected and segmented into objects. Here, cell outlines were used as regions of interest. Secondly, the normalized Ripley’s K function namely L(r) was calculated on previously segmented objects for each cell with a minimum radius of 10 nm, maximum radius of 200 nm and step radius of 10 nm. L(r) of all cells were averaged and presented in figures as mean ± SD. Means of L(r) between experimental groups were statistically compared using either Student’s t-test or ANOVA in Prism software (Graphpad, US).

### GLUT4 molecule density analysis

dSTORM images contain millions of molecule coordinates and subtle changes in parameters such as molecule density of the previously obtained localizations and clustering cannot be qualitatively assessed. Here, we have used the Fiji Macro LocFileVisualizer_v1.1 to quantify plasma membrane molecule densities^39^. In brief, upon execution of LocFileVisualizer the operation ‘Import&Generate’ was selected with gray value quantification activated, a localization file selected and the desired lateral pixel size (20 nm) specified to obtain a rendered image of molecule coordinates. The Fiji ‘Polygon selections’ tool was used to draw around the cell outline and the outline was saved in the ‘ROI manager’. This process was repeated for every cell in the data set. The Fiji ‘Measure’ function was applied to quantify ‘Integrated density’ of GLUT4 and TfR molecules within each cell. Prism software v9 (Graphpad, CA, USA) was used to normalize obtained molecule density against area for each cell and facilitate visualisation of results. It should be noted that by the nature of the dSTORM/TIRF imaging, we did not capture total GFP expression signals from the cells studied and thus cannot correct the translocation data for total GLUT4 expression in this dataset.

## Results and discussion

### Insulin-stimulated GLUT4 dispersal occurs in many cell types

**Figure 1:**
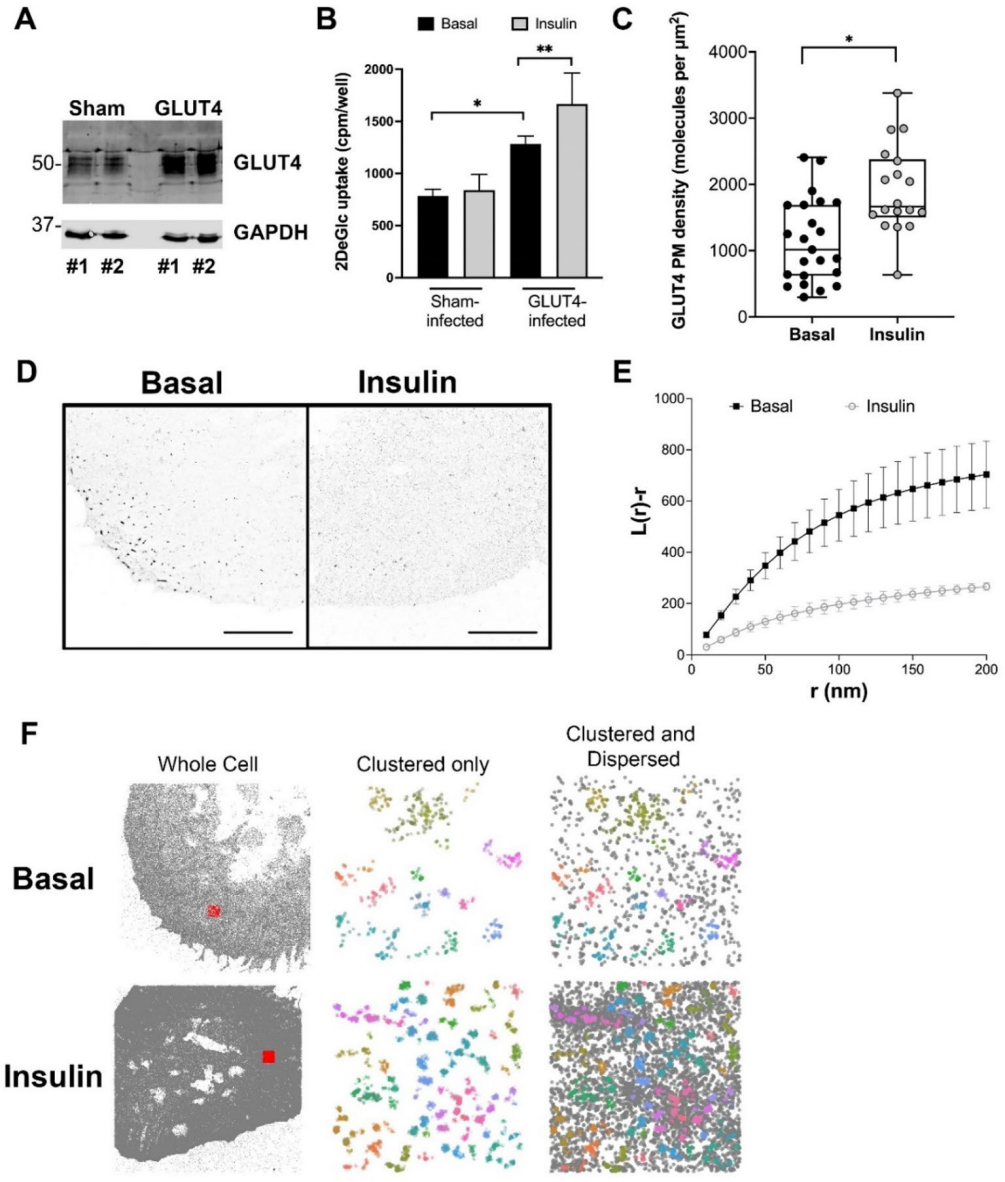
Behaviour of GLUT4 in iPSC-cardiomyocytes. (**A**) We over expressed human GLUT4 using lentivirus in iPSC-CMs. Shown are two representative examples (#1 and #2) of empty virus infected cells (‘Sham’) and cells infected with GLUT4 virus (‘GLUT4’). Lysates were immunoblotted for GLUT4 and GAPDH and increased levels of GLUT4 are evident in GLUT4-virus infected cells. Levels of over-expression ranged from 2- to fourfold between experiments. (**B**) Deoxyglucose (DeGlc) uptake was assayed in the presence and absence of insulin in parallel batches of virus infected cells. Data shown is from three independent biological replicates. GLUT4 overexpression increased basal glucose transport (*p = 0.01), and cells overexpressing GLUT4 exhibited insulin-stimulated glucose transport (**p = 0.05). Sham-infected cells did not exhibit insulin-stimulated glucose transport. Statistical analysis was performed using an unpaired t-test on raw data. (**C**) We expressed HA-GLUT4-GFP in iPSC-CMs and quantified localisation density of cell surface GLUT4 (as HA staining) before and after stimulation with 100 nM insulin for 20 min. The data from replicate experiments is presented as a box and whiskers plot, each point represents data from a single cell. Insulin stimulated a 1.7-fold increase in cell surface HA staining, *p = 0.0005 by unpaired two sample t-test. (**D**) Representative images of iPSC-CMs expressing HA-GLUT4-GFP stained for HA as outlined. Scale bars = 10 μm. (**E**) GLUT4 molecule coordinates were obtained using ThunderSTORM and their spatial pattern was analysed using normalized Riley’s K function (L(r)), derived from the Voronoï diagram of GLUT4 molecule coordinates. L(r) describes how many segmented clusters can be found within a distance r of any arbitrary point. Empirical estimates of L(r) are shown for basal and insulin-stimulated cells. The experiment was carried out independently on > n = 10 cells per group and the basal and insulin values differed significantly (p < 0.0001; determined by unpaired two samples t-test). (**F**) GLUT4 molecule coordinates were processed using a python *HDBSCAN* script written in house. Gray dots indicate single molecule localizations for representative whole cells (left panels). Representative 4 μm × 4 μm ROIs are highlighted by the red squares. The middle panels show coloured molecule clusters identified by *HDBSCAN* (‘clustered only’) and coloured clusters plus gray non-clustered molecules (‘clustered and non-clustered’, right panels) within representative ROI.

All studies of the spatial distribution of GLUT4, including work from our group using dSTORM, have thus far been performed in 3T3-L1 adipocytes^9,18,20,33^. To extend this, we examined whether this dispersal could also be observed in other cell types and focussed first on another insulin-sensitive cell type—cardiomyocytes. We used induced pluripotent stem cell-derived cardiomyocytes (iPSC-CMs), cells which have previously been used as a model of insulin action in cardiomyocytes^40^. These cells were chosen as attempts to overexpress HA-GLUT4-GFP in primary cardiomyocytes proved unsuccessful. Naïve iPSC-CMs express relatively little GLUT4 and do not exhibit insulin-stimulated glucose transport as GLUT1 levels are significantly higher than GLUT4^28^. We first overexpressed GLUT4 in these cells to verify that they exhibit insulin-stimulated glucose transport. We used a lentivirus to drive over-expression of human GLUT4. In multiple experiments of this type (shown in Fig. 1A), a 2- to fourfold increase in GLUT4 levels was routinely observed. In parallel, overexpression of GLUT4 elevated basal 2-deoxyglucose uptake and reconstituted insulin-stimulated glucose transport in these cells (Fig. 1B), providing confidence in iPSC-CMs as a model^28,40^. We therefore expressed HA-GLUT4-GFP in these cells and quantified GLUT4 (as HA) surface density. dSTORM images acquired using this approach contain millions of molecule coordinates allowing changes in parameters such as molecule density to be quantified. Here, we have used the Fiji Macro LocFileVisualizer_v1.1 to quantify plasma membrane molecule densities^39^ which revealed an insulin-stimulated translocation of HA-GLUT4-GFP to the cell surface of 1.7-fold (*p* = 0.0005) (Fig. 1C), a value in broad agreement with studies in primary cardiomyocytes^41^.

We then used dSTORM to test whether insulin promotes dispersal of GLUT4 within the plasma membrane of iPSC-CMs (representative images are shown in Fig. 1D). Two independent methods were employed to illustrate the effect of insulin. We used SR Tesseler software to generate Voronoï diagrams of molecule coordinates which were used to compute normalized Ripley’s K-functions namely L(r) (Fig. 1E). As shown, L(r) peaks at higher values for basal cells indicating more GLUT4 is found clustered at closer distances compared to insulin-stimulated cells, and closely resembles that reported by others^20^. In agreement with this conclusion, qualitative representations derived from HDBSCAN analysis (Fig. 1F) indicate that insulin increases both the density of GLUT4 clusters (shown as coloured regions) and the amount of dispersed GLUT4 (shown as black dots) in the plasma membrane of these cells. These data support the hypothesis that insulin-stimulated GLUT4 dispersal is exhibited in other insulin-sensitive cell types.

GLUT4 exhibits insulin-dependent translocation when expressed in HeLa cells with properties similar to that observed in other cell systems^26,42,43^. We therefore chose this cell line to compare the behaviour of over-expressed GLUT4 and transferrin receptors (TfR) because of their ease of transfection (transfection efficiencies are very low in both 3T3-L1 adipocytes and iPSC-CMs). TfR populate the general recycling system, and exhibit insulin-stimulated redistribution to the surface of adipocytes^44^. Comparison between GLUT4 and TfR trafficking and translocation kinetics has revealed a clear distinction, with the idea that TfR accesses the cell surface from endosomal compartments and GLUT4 from specialised GSC^30,45–48^. We therefore sought to compare the magnitude of insulin-stimulated translocation of GLUT4 and TfR in HeLa cells, and to ask whether differences in dispersal could be observed in response to insulin.

**Figure 2:**
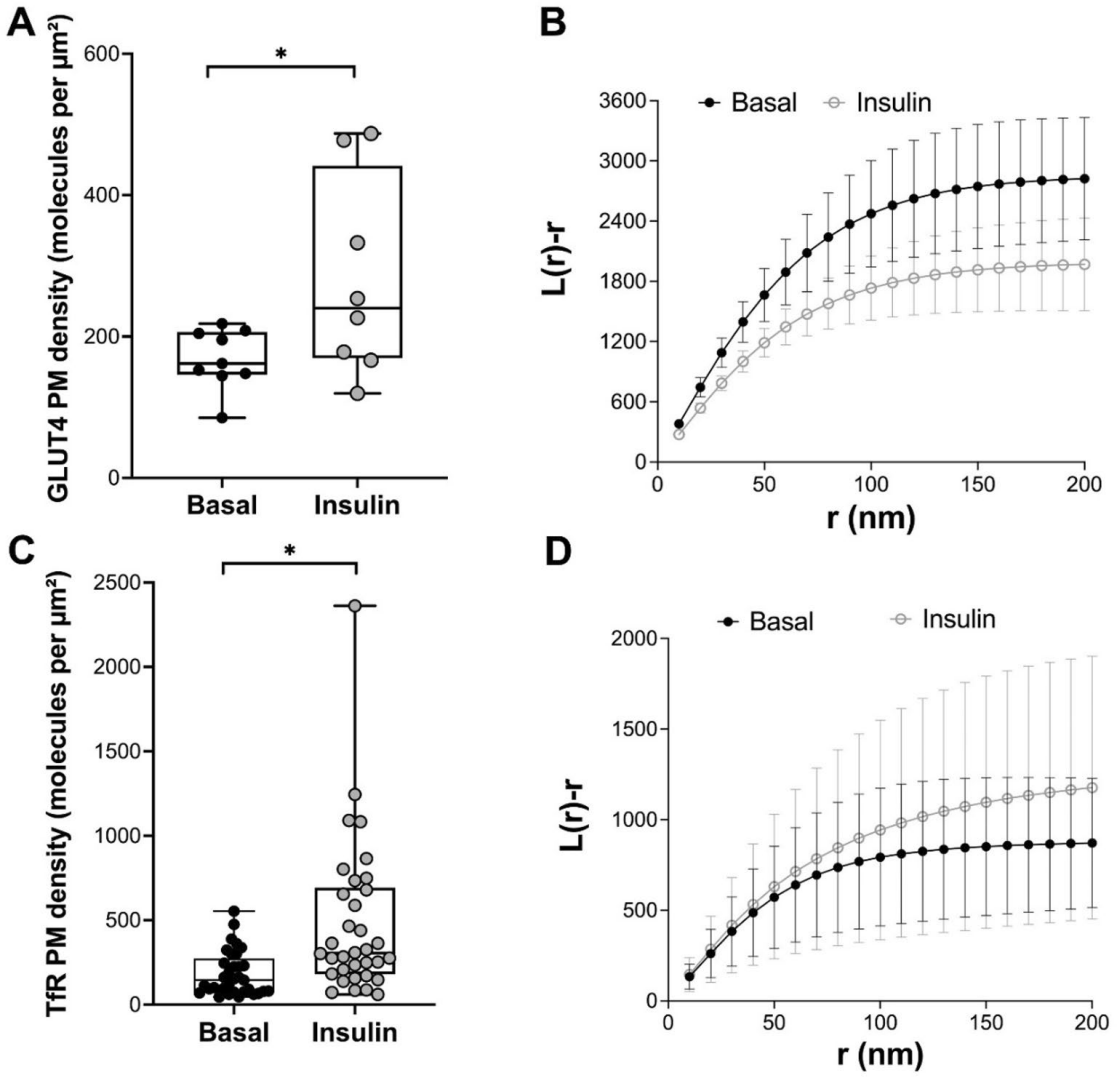
Behaviour of GLUT4 and TfR in HeLa cells. HeLa cells expressing HA-GLUT4-GFP were incubated in serum free media for 2 h, followed by insulin challenge (1 μM insulin for 20 min). Cells were fixed and the HA epitope was stained for dSTORM as outlined. (**A**) shows quantification of the density of cell surface GLUT4 (as HA staining) before and after insulin, presented as the merged data from at least 8 cells from 3 biological replicates (*p = 0.0007 determined by unpaired two samples t-test); each point is a measurement from a single cell. (**B**) GLUT4 molecule coordinates were obtained using ThunderSTORM. Empirical estimates of L(r) were calculated from the Voronoï diagrams of GLUT4 molecule coordinates under basal and insulin-stimulated conditions. The experiment was carried out independently on > 8 cells per group from 3 biological replicates and revealed a statistical difference between basal and insulin-stimulated cells (p = 0.0028; determined by unpaired two samples t-test). HeLa cells expressing TfR-GFP were used in a similar analysis. (**C**) shows quantification of TfR surface density before and after insulin treatment, presented as the merged data from > 30 cells from > 3 biological replicates (*p = 0.0005 determined by unpaired two samples test); each point is a measurement from a single cell. TfR molecule coordinates were obtained using ThunderSTORM and (**D**) shows estimates of L(r) under basal and insulin-stimulated conditions. No difference between basal and insulin-stimulated conditions was observed (p = 0.0683; determined by two-samples t-test).

Consistent with previous studies, both GLUT4 (Fig. 2A) and TfR (Fig. 2C) exhibited increased density at the cell surface after insulin treatment. Ripley’s K-function analysis revealed a significant difference in control and insulin-stimulated cells for GLUT4, consistent with insulin-stimulated dispersal (Fig. 2B; also see above). By contrast, insulin treatment did not result in a similar change in the dispersal of TfR (Fig. 2D). These data suggest that the insulin-stimulated dispersal of GLUT4 is an intrinsic property of GLUT4 and is not shared by all recycling plasma membrane-localised proteins.

### Insulin-stimulated GLUT4 dispersal is impaired in models of insulin resistance

**Figure 3:**
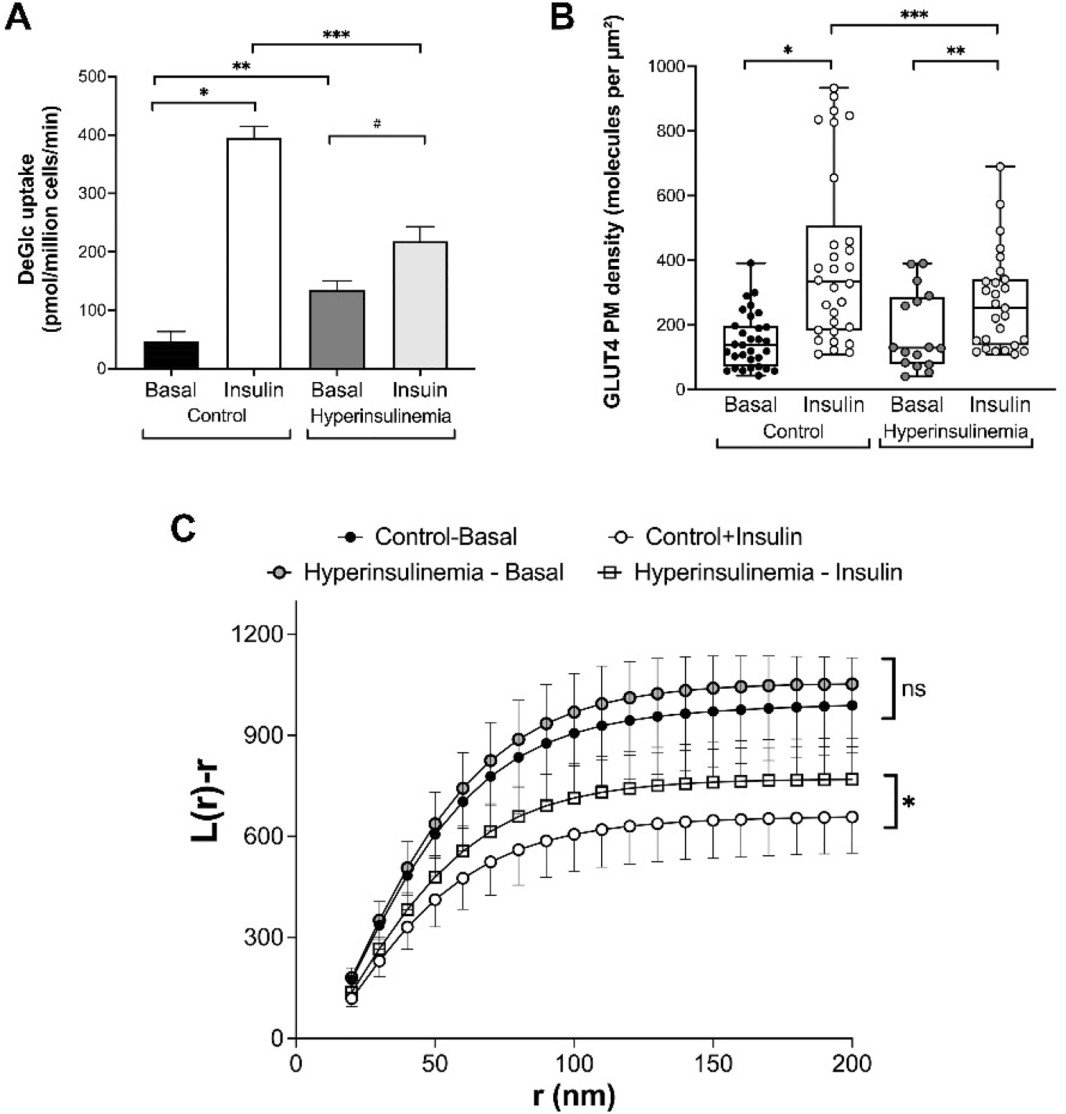
Chronic insulin treatment modulates GLUT4 translocation and dispersal. 3T3-L1 adipocytes were incubated in normal media or in media supplemented with 500 nM insulin for 24 h (‘hyperinsulinemia’ in the figure) before washing as described in *Methods*. (**A**) shows the rates of 2-deoxyglucose (DeGlc) transport in response to an acute insulin challenge (100 nM, 20 min); insulin robustly stimulates glucose transport in control cells (8.4-fold; *p < 0.0001). Hyperinsulinemia results in elevated basal (insulin-independent) DeGlc uptake (2.8-fold, **p = 0.0002) and impairs the response to an acute insulin addition (reduced to 1.6-fold; #*p* = 0.0013). The maximal rate of insulin stimulated DeGlc transport was reduced under hyperinsulinemia (from 394 pmol/min/million cells to 218 pmol/mon/million cells; ***p < 0.0001). Data shown is from 4 independent experiments; statistical analysis by one-way ANOVA. We used 3T3-L1 adipocytes expressing HA-GLUT4-GFP to measure GLUT4 plasma membrane density (**B**) and GLUT4 dispersal (**C**) under the same conditions. (**B**) Insulin increased GLUT4 density in the plasma membrane 2.7-fold (*p < 0.0001) in control cells. In chronic insulin treated cells, the ability of insulin to increase GLUT4 levels was reduced (1.5-fold, **p = 0.044); each point is a measurement from a single cell. The maximal GLUT4 density in response to insulin was reduced in chronic insulin treated cells (***p = 0.03). (**C**) shows estimates of L(r) under basal and insulin-stimulated conditions in control and hyperinsulinemia conditions as previously described. Chronic insulin treated cells and control cells in the absence of insulin exhibited no difference in L(r) distribution (ns on figure; *p* = 0.86 determined by one-way ANOVA, Tukey’s HSD Test). Acute insulin stimulation resulted in GLUT4 dispersal in both control and chronic insulin-treated cells. However, chronic insulin treated cells exhibited reduced GLUT4 dispersal in response to an acute insulin challenge compared to that observed in control cells (*p = 0.0061 determined by one-way ANOVA, Tukey’s HSD Test).

Previous work from others has suggested that the insulin-simulated dispersal of GLUT4 is impaired in models of insulin resistance^20^. To substantiate this, we incubated 3T3-L1 adipocytes with insulin overnight, a treatment previously shown to render the cells refractory to further insulin challenge^31,32^ and verified that this treatment rendered the cells refractory to acute insulin-stimulated glucose transport (Fig. 3A).

We then analysed both plasma membrane density and dispersal of GLUT4 using dSTORM under these conditions. Chronic insulin treatment reduced the plasma membrane density of GLUT4 in parallel with the observed changes in glucose transport (Fig. 3B); it is of note that the changes in GLUT4 density measured using this approach are qualitatively similar to those generated using cell surface-specific GLUT4 photo-labels^31^, further validating this approach. Analysis of the same dSTORM dataset (Fig. 3C) revealed that L(r) peaks at higher values for basal cells indicating more GLUT4 is found clustered at closer distances. Chronic insulin treatment did not change this distribution significantly. By contrast, while acute insulin stimulation resulted in a reduction of the L(r) peak in both control and chronic insulin treated cells, this effect was significantly impaired in cells exposed to hyperinsulinemia. Thus, our data is consistent with previous studies indicating that insulin resistance is accompanied by a reduction in GLUT4 dispersal in 3T3-L1 adipocytes^20^.

### GLUT4 translocation and dispersal are cell-size-dependent phenomena

Adipocytes, including cultured 3T3-L1 adipocytes, are known to exhibit considerable heterogeneity. This is manifest at the level of different gene expression patterns and rates of glucose accumulation^49,50^, rate of differentiation and lipid droplet accumulation^51^ and in metabolic phenotypes^52–54^. This is of significance as primary adipocytes exhibit both hypertrophy and hyperplasia with corresponding metabolic disturbances, e.g., patients with type-2 diabetes exhibit hypertrophy^54–56^. In line with this, studies have suggested that larger adipocytes exhibit reduced insulin-stimulated glucose transport and reduced levels of GLUT4 translocation to the cell surface. These studies were, however, limited to analyses of cell populations. More recently we have used a glucose biosensor coupled to novel data analysis programs to show heterogeneity in insulin-stimulated glucose transport in 3T3-L1 adipocytes which range in size from 10 to 150 μm^49^. Consistent with these studies we observed that larger 3T3-L1 adipocytes were less insulin-sensitive than their smaller counterparts. Cultured 3T3-L1 adipocytes therefore exhibit some of the properties associated with cell size that have been identified in primary cells which also exhibit a wide range of size heterogeneity (values between 20 and 300 μm were reported for human adipocytes^25,54,57–59^).

**Figure 4:**
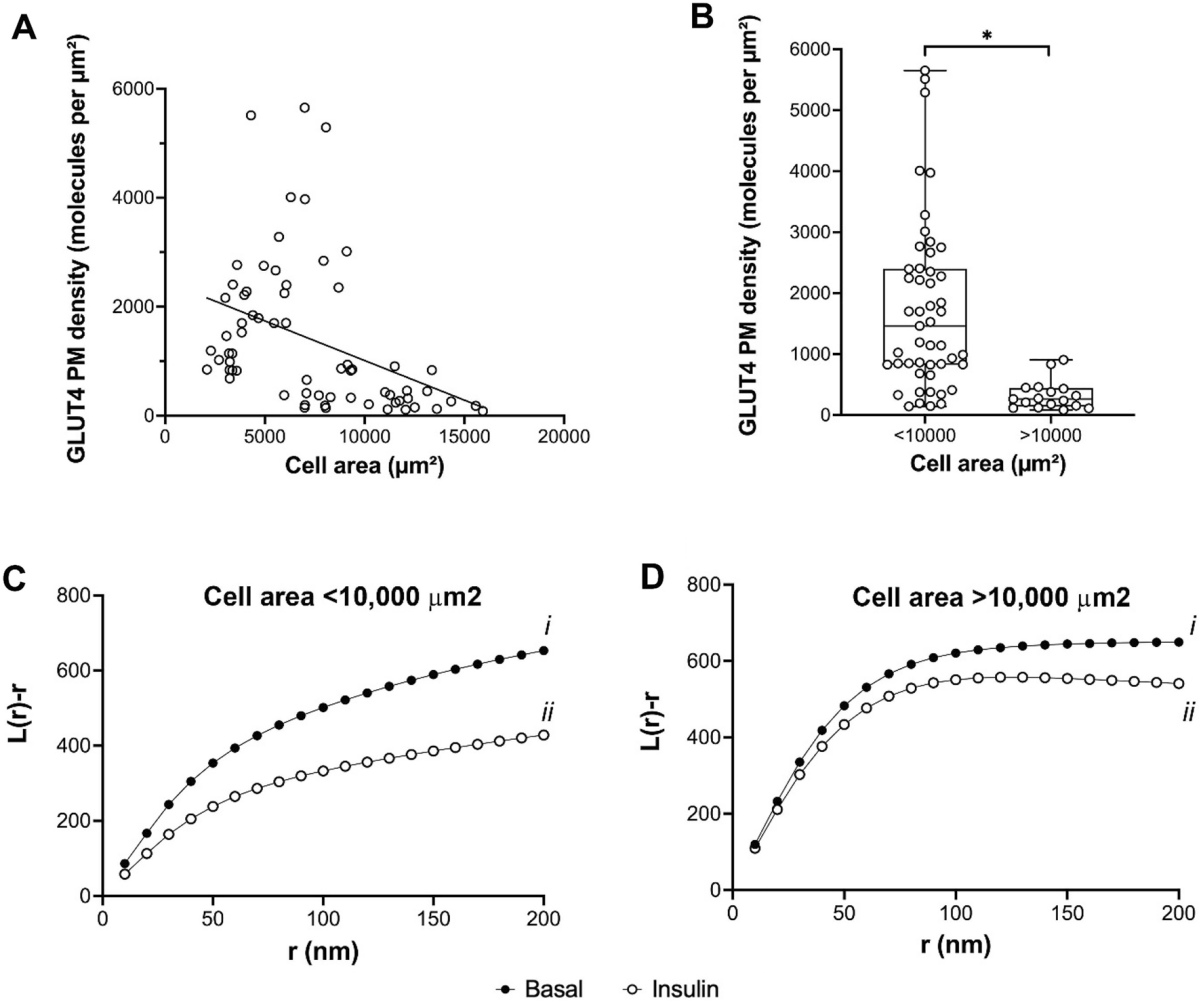
GLUT4 translocation and dispersal in adipocytes of different sizes. dSTORM datasets were used to quantify cell surface GLUT4 density in cells incubated with or without 100 nM insulin for 20 min as outlined, and the data sorted on the basis of cell size. (**A**) shows the relationship of GLUT4 density to cell area in insulin-stimulated cells (100 nM, 20 min). Each point is a single cell, and the data was collected from 70 to 150 cells analysed from 3 independent experiments. (**B**) shows the same data sorted into cells of area less than or greater than 10,000 μm^2^. *Indicates a statistically significant difference between the groups, *p* = 0.001. Each point is a measurement from a single cell. (**C**) and (**D**) show estimates of L(r) under basal and insulin-stimulated conditions separated on the basis of cell size. (**C**) shows that in cells of area < 10,000 μm^2^, L(r) peaks at higher values for basal cells indicating more GLUT4 is found clustered at closer distances compared to insulin-stimulated cells (*p* = 0.008 for curves i versus ii).), consistent with insulin-stimulated GLUT4 dispersal (see text). (**D**) indicates the ability of insulin to promote dispersal is significantly impaired in larger cells (*p* = 0.132 for curves i versus ii). Analysis of the basal distribution shows no significant difference between smaller and larger cells (Ci vs. Di, *p* = 0.121), but a significant difference in insulin-stimulated distribution (Cii versus Dii, *p* < 0.0001). All statistical comparisons were determined by unpaired two-samples t-test.

We therefore quantified GLUT4 translocation and dispersal in 3T3-L1 adipocytes, sorted on the basis of size, using our dSTORM datasets. We first explored the relationship between GLUT4 plasma membrane density in insulin-stimulated cells with cell size (Fig. 4A).

These data reveal a negative correlation between cell area and the density of GLUT4 molecules on the surface after insulin treatment. This is emphasised when the data are sorted into cells less than or greater than 10,000 μm^2^ (Fig. 4B). Thus, larger cells deliver less GLUT4 to the surface, on average, than their smaller counterparts. Figure 4B implies a roughly fivefold higher density of GLUT4 in smaller adipocytes than larger cells. These data are consistent with reduced insulin-stimulated GLUT4 translocation reported in larger fat cells^60,61^, studies of hypertrophic adipocytes^62,63^ and our data on glucose transport in 3T3-L1 adipocytes of different sizes^49^. This effect would be magnified if the reported inverse relationship between adipocyte size and GLUT4 levels in human adipocytes^64^ also operates in 3T3-L1 adipocytes. Such changes in translocation likely underpin the observed differences in cell surface GLUT4 levels in human adipocytes isolated from subjects with differing degrees of insulin sensitivity^24,65^. Interestingly, our data on GLUT4 dispersal, when separated into similar populations (Fig. 4C,D), suggests that the extent of GLUT4 dispersal is also greater in smaller cells (Fig. 4C) compared to larger cells (Fig. 4D). These studies are limited by two important caveats. First, we have no ready mechanism to assess overall HA-GLUT4-GFP expression levels in the individual cells examined, and thus cannot directly correlate HA levels at the surface with total cellular HA-GLUT4-GFP expression. dSTORM images were acquired in TIRF mode which is an elegant method that uses an evanescent wave to only illuminate and excite selected fluorophores immediately adjacent to the coverglass. This is highly advantageous when imaging surface regions such as the basal plasma membrane because axial resolution is considerably improved by elimination of signal from fluorophores in the background. However, this technique is at a disadvantage when it comes to assessment of total GLUT4 expression within the cells. Acquisition of diffraction limited images in TIRF or standard epifluorescence illumination to capture total GFP expression within each cell is possible but would still make it difficult to normalize against the HA-staining signal from the basal plasma membrane alone. Secondly, we recognise the limitation in extrapolating data from cultured differentiated 3T3-L1 adipocytes to primary cells which likely exhibit distinct regulatory mechanisms and are subject to paracrine and autocrine controls not evident in culture. Studies in of GLUT4 dispersal in primary cells are clearly warranted.

These caveats notwithstanding, our data support the hypothesis that the dynamics of GLUT4 on the surface of cells is correlated with transport activity and may underpin elements of insulin resistance observed in adipocytes of different sizes.

## Conclusions

Our data are consistent with the idea that insulin-stimulated GLUT4 delivery to the plasma membrane of adipocytes, iPSC-CMs and HeLa cells is accompanied by dispersal from a clustered to a more diffuse distribution, and thus argue that this is an inherent property of GLUT4. Consistent with this, dispersal of transferrin receptors in Hela cells is not changed by acute insulin challenge. Insulin-stimulated GLUT4 dispersal is impaired in a model of insulin resistance, suggesting that this dispersal could play a role in the development of insulin resistance. It is also worth noting that the capacity for insulin-stimulated GLUT4 dispersal is ‘encoded’ within GLUT4 and is not specific to cells that normally express this transporter. Finally, we show that both cell surface levels of GLUT4 and GLUT4 dispersal are negatively correlated with adipocyte size, supporting previous arguments that larger adipocytes are less insulin responsive than their smaller counterparts.

## Supplementary Information

Supplementary Information.

## Data Availability

The datasets used and/or analysed during the current study are available from the corresponding authors on reasonable request.
